# Low-dose oral hydroxychloroquine led to impaired vision in a child with renal failure: Case report and literature review

**DOI:** 10.1097/MD.0000000000024919

**Published:** 2021-03-12

**Authors:** Jinmiao Lu, Yidie Huang, Qiaofeng Ye, Feineng Shang, Mei Ming, Hong Xu, Zhiping Li

**Affiliations:** a Department of Clinical Pharmacy, Children's Hospital of Fudan University, Shanghai; b Department of Pharmacy, Dehong People's Hospital, Yunnan; c Department of ophthalmology, Huangshi Central Hospital, Affiliated Hospital of Hubei Polytechnic University, Huangshi; d Department of Nephrology, Children's Hospital of Fudan University, Shanghai, China.

**Keywords:** decreased visual acuity, hydroxychloroquine, lupus nephritis, renal hypofunction

## Abstract

**Introduction:**

Hydroxychloroquine (HCQ) has received much attention in the treatment of coronavirus disease 2019 recently. However, it can cause irreversible vision loss. Few cases have been reported in pediatric patient with HCQ-related adverse reactions. Appropriate administration and early disease recognition are important for reducing the adverse drug reactions of HCQ.

**Patient concerns:**

We report a case of a 14-year-old Chinese girl who sought treatment for rapidly decreasing vision in the left eye over 3 days. The simulation results of the population pharmacokinetic model of HCQ revealed that the plasma concentration of HCQ abnormally increased before the visual acuity of the eye decreased.

**Diagnosis:**

She was diagnosed as HCQ related drug adverse reaction.

**Interventions:**

The daily dose of HCQ for this patient was adjusted from 100 mg/d to 50 mg/d.

**Outcomes:**

Follow-up for 6 months showed no more vision loss recurrence. However, the existing decreased visual acuity of the eye did not recover either.

**Conclusion:**

Although decreased visual acuity is an infrequent symptom, ophthalmologists should be aware of the possibility of HCQ concentration enrichment and consider minimizing HCQ use when a child with renal hypofunction seeks treatment for shortsightedness.

## 1 Introduction

The chemical structure of hydroxychloroquine (HCQ) is similar to that of chloroquine (CQ). However, HCQ is considered better tolerated than CQ. Furthermore, HCQ is less toxic than CQ; however, its elimination half-life in humans, which can be as long as 52 days, is noteworthy. The use of HCQ in patients with systemic lupus erythematosus can reduce disease activity and recurrence, organ damage, and lupus-related renal failure and improve survival. Many current treatment guidelines for lupus nephritis (LN) recommend HCQ as the basic treatment for LN. HCQ retinal toxicity and the duration of HCQ use and daily HCQ dose are related to the presence or absence of kidney disease.^[1]^ In fact, in the case of renal insufficiency, more stringent monitoring of HCQ use should be considered; however, there are very few relevant studies.

HCQ is among the proposed drugs and is the most widely used for coronavirus disease 2019 management, despite the lack of robust evidence on its effectiveness.^[2]^ The long-term use of HCQ is related to eye-related diseases, such as retinopathy and macular degeneration. ^[3]^ However, the related underlying mechanism remains unclear. HCQ-induced retinopathy can lead to irreversible loss of central vision, which can progress even if the patient has stopped taking the drug. Therefore, appropriate administration methods and recognition of early ocular lesions are crucial for reducing adverse visual sequelae among HCQ-treated patients. Although HCQ-induced vision loss is common, there is no literature available on the long-term low-dose application of HCQ in children with LN showing that it causes adverse ophthalmic reactions. Herein, we describe a case of a child with chronic kidney disease who showed adverse eye reactions after using low-dose HCQ for 20 months. To determine the cause, therapeutic drug monitoring was conducted and HCQ blood concentrations were analyzed using pharmacokinetic approaches.

## 2 Case presentation

A case of an ophthalmic adverse reaction caused by long-term low-dose HCQ use was reported. The patient was a 14-year-old child of Han nationality. She weighed 38 kg, had been diagnosed with LN 7 years ago, and was receiving HCQ 100 mg daily. The average daily dose of HCQ in this child was 2.6 mg/kg/d, approximately half of the maximum recommended safe daily dose (≤5.0 mg/kg/d) of HCQ to preventetinal toxicity. The patient had been using HCQ for 20 months since September 16, 2018, when the adverse ophthalmic reactions occurred. This study was approved by the ethics committee of each individual children's hospital? (NO. 2020-187) and followed the Declaration of Helsinki.

### 2.1 Ophthalmic testing

**Figure 1:**
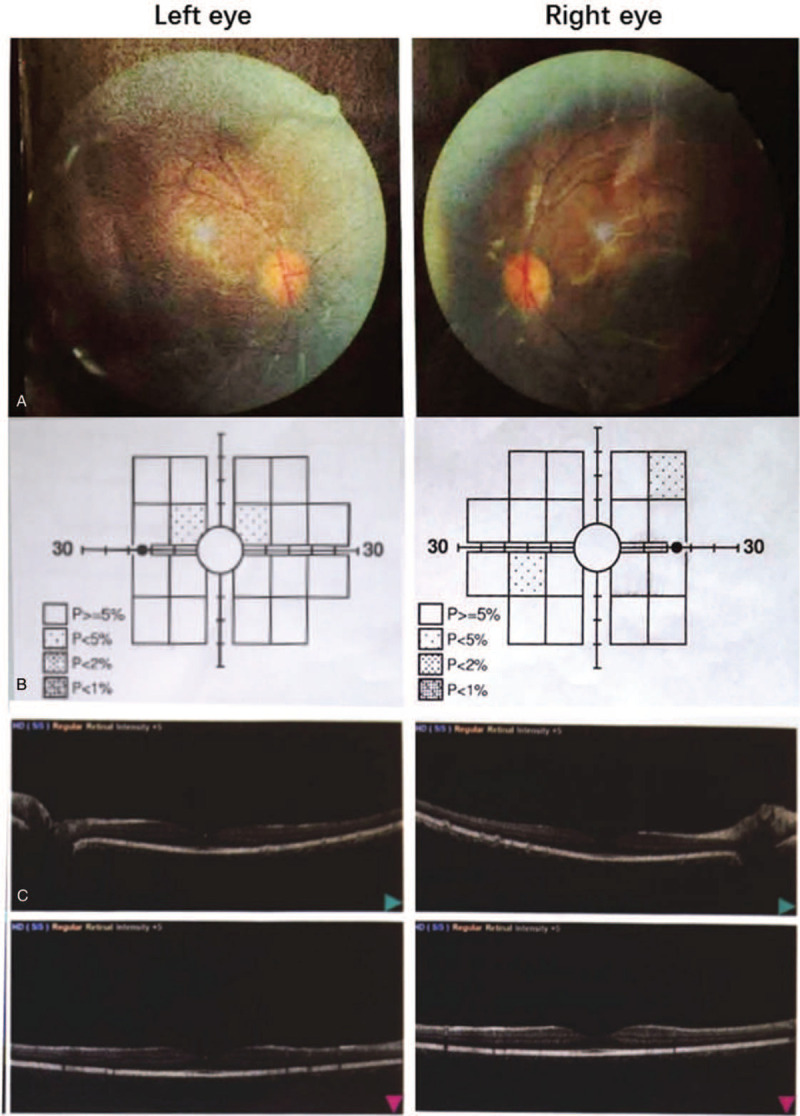
Photographic and fundus film examination of both eyes of the patient (A); visual field examination of both eyes (B); optical coherence tomography (OCT) graphs of the eyes (C).

An eye examination performed on May 20, 2020, revealed the following findings: visual acuity, left 1.0 and right 1.0; intraocular pressure, left 19 mm Hg and right 17 mmHg; corneal thickness, left 555 μm and right 544 μm; and bright corneas, clear anterior chambers, and round pupils of both eyes. Both eyes reflected light, and each lens was transparent; fundus photography showed no apparent abnormality. Optical coherence tomography revealed that the shape of the fovea in both eyes was standard; there was no evidence of organic eye disease. However, the central visual field examination revealed slightly decreased local visual acuity of both eyes, of which 67% of the fixation losses were in the left eye; no damage was noted in the right eye (Fig. 1).

### 2.2 Medical history

The general dose of HCQ for children is 5 mg/kg/d, and the maximum daily dose should not exceed 200 mg. If the glomerular filtration rate is <30 ml/min/1.73 m^2^, the dose needs to be adjusted. In the case of this patient, the HCQ dose was reduced considering the child's initial estimated glomerular filtration rate (eGFR) was 26 ml/min/1.73 m^2^. On the other hand, from the first HCQ administration to May 20, 2020, the cumulative HCQ dose was 61.2 g, which was far lower than the standard risk dose of 1000 g. Therefore, the child should not have presented with adverse ophthalmic reactions. However, the adverse reactions of long-term HCQ use include macular degeneration, macular disease, retinal pigment changes, and visual field defects. Therefore, the slight decrease in the child's visual acuity was attributed to her HCQ use, and we primarily suspected that the adverse ocular reactions in the child were a result of a high HCQ concentration. We retrospectively evaluated the patient's drug concentration levels in different periods after HCQ treatment by using previously frozen blood samples.

### 2.3 Serum concentration monitoring

High-performance liquid chromatography-tandem mass spectrometry was used to detect the blood concentration of HCQ. Hydroxychloroquine concentration in plasma was determined by high-performance liquid chromatography–tandem mass spectrometry method.^[4]^ Chromatograph was carried out on a Kinetex C18 column. The mobile phase was 0.6%formic acidwater (pH3.2)-methyl alcohol (80:20). The flow rate was set at 0.5 ml min^−1^. Column temperature was 25 °C. The retention time of HCQ and internal standard CQ were approximately 1.3 min and 1.5 min, respectively. A good linearity was shown in concentration range 0.200 to 100 μg·L-1 for HCQ. The LLOQ was 0.2 μg·L-1. The method recovery was 98.20% to 102.75%.

### 2.4 Population pharmacokinetic simulation

**Figure 2:**
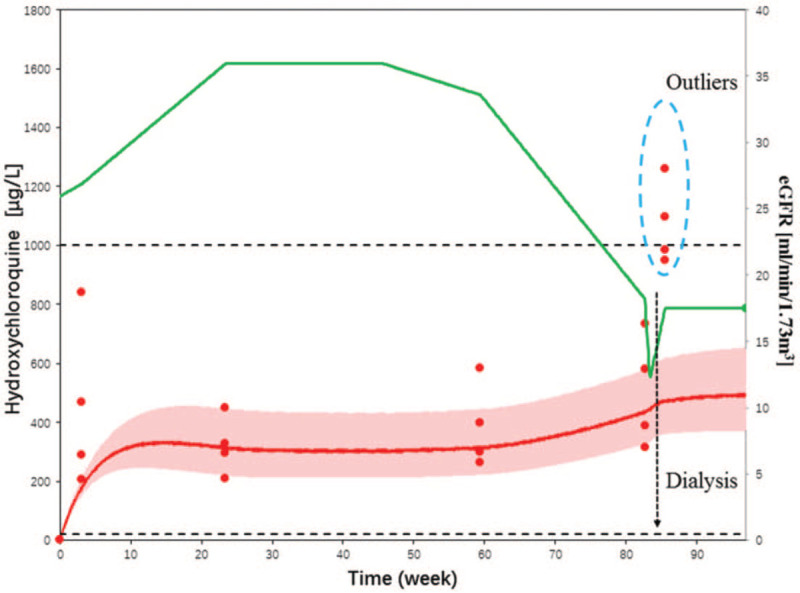
Population pharmacokinetic curve of the blood concentration of hydroxychloroquine. The red line represents the population pharmacokinetic simulation curve for hydroxychloroquine. The red shade represents the 95% confidence interval. The red dots represent the monitored concentration of hydroxychloroquine. The green line represents the estimated glomerular filtration rate (eGFR) of the patient during the same period.

Blood samples were collected during four hospital stays (Fig. 2). The blood sampling time points were 30 min before HCQ administration and 20, 60, and 120 min after HCQ administration. The blood sample was a collected test sample of mycophenolate mofetil, frozen in a refrigerator at −80°C after the test. After performing simulations on all blood drug concentrations with a population pharmacokinetic model in MwPharm, it was found that the trough HCQ concentration remained low for a long time when HCQ was used at low doses. Although the trough HCQ concentration increased slightly with a decrease in renal function, the change was not significant, and the long-term dose range of HCQ was still below the recommended level (1000 μg/L). However, with the progression of kidney disease, the child's HCQ concentration increased sharply after receiving dialysis. Shortly thereafter, her visual acuity decreased. As is known, HCQ is excreted through the kidneys, with a half-life of up to 52 days. HCQ accumulates in the tissues and cannot be excreted from the body through hemodialysis or other methods. Therefore, we inferred that dialysis cannot remove HCQ in time; nevertheless, dialysis may cause the release of HCQ from the tissues into the blood, resulting in a sharp increase in its concentration, which induces adverse ophthalmic reactions. Thus, for patients with severely impaired renal function, plasma HCQ levels should be monitored to adjust the dose after dialysis.

According to “The Renal Drug Handbook,” HCQ cannot be removed from the body by hemodialysis.^[5]^ The book points out that when the GFR is <10 mL/min, that is, the dose of HCQ is 50 to 100 mg daily, the drug should be used with caution. Finally, since this patient started dialysis and had a weight lower than normal, HCQ reduced to 50 mg daily. Follow-up for 6 months showed no clinical or radiological evidence of disease recurrence. However, the visual acuity of the eye decreases did not recover either.

## 3 Discussion

The overall prevalence of HCQ-induced retinopathy is 7.5%, and its clinical manifestations are usually described as bilateral bull's eye macular degeneration.^[6]^ A patient is not diagnosed with retinopathy until there are noticeable peripheral changes, peripheral pigment changes, weakening of retinal blood vessels, optic nerve atrophy, peripheral visual field limitation, and an abnormal electroretinogram. Typical ”bull's eye" changes are not visible.^[7]^ Therefore, the current guidelines recommend screening before damage to the retinal pigment epithelium, and HCQ toxicity can be determined through imaging or fundus examination. However, such preventive measures are not always effective.^[8]^ HCQ retinopathy appears to be significantly related to the duration of use, cumulative HCQ dose, and renal function.^[9]^ Among them, decreased renal function, as an independent influencing factor, may lead to slower HCQ excretion and secondary HCQ retinal toxicity.^[10]^ This patient developed LN secondary to systemic lupus erythematosus. After two years, the eGFR level in the kidneys gradually decreased and the trough level of HCQ in her body gradually increased. It is worth pointing out that when a patient undergoes dialysis because of a sharp decrease in eGFR, the HCQ level in the body increases sharply (>1000 μg/L) and the adverse reaction of decreased visual acuity occurs 1 week after dialysis.

Visual acuity is usually good in the early stages of HCQ treatment until the severe injury stage, and most patients with HCQ toxicity have no visual symptoms. Vision and visual acuity are mainly controlled by cells in the macular area. If patients with macular degeneration continue to be exposed to HCQ, macular degeneration invades the central portion and eventually causes vision loss.^[11]^ In another study, decreased sensitivity of the fovea in each eye was noted in a 15-year-old patient who had been using HCQ for 5 years. The other checks did not show any changes, the cumulative dose was only 353 g. At 7 years of HCQ treatment, bilateral inferior central macular thinning could be seen on optical coherence tomography, suggesting bull's eye macular degeneration.^[12]^ The findings in this study were consistent with those in our patient. This indicates that decreased visual acuity in children may be a precursor to retinopathy. Related phenomena such as macular degeneration and retinopathy, which were considered to be the early symptoms of retinopathy, were not found in the child.

Control of HCQ concentration is a critical factor in the regulation of ophthalmological diseases. In a study of 1556 patients using HCQ, no retinal toxicity was observed in patients treated with HCQ at a daily dose of <6.5 mg/kg/day. It is possible that the use of low-dose HCQ reduced the progression of retinopathy.^[13]^ In contrast, high-dose HCQ can lead to earlier development of retinal toxicity. A study of 2361 patients who had used HCQ for more than 5 years found that when the cumulative dose exceeded 1000 g of HCQ, the risk of ocular toxicity significantly increased.^[14]^ At a dose of 6.5 mg/kg, the risk of retinal toxicity within 5 and 10 years is less than 1% and 2%, respectively.^[15]^ In addition, the deposition of HCQ in the cornea could decrease visual acuity.^[16]^ Patients with long-term use of HCQ showed lower corneal endothelial cell density and higher central corneal thickness than did the control group.^[17]^ The corneal deposit changes are dose-dependent, transient, and reversible. In this patient, no corneal deposition was found. The reason for this could be that the child's blood concentration had been maintained at a low level in the early treatment period and the HCQ concentration suddenly increased at a later point, resulting in unobvious symptoms. Similarly, previous case studies found that HCQ-induced vortex keratopathy is dose- and duration-dependent.^[18]^

Studies have shown that despite discontinuation of HCQ treatment, eye diseases continue to develop.^[19]^ Therefore, it is necessary to further investigate the pathogeneses of these diseases. The mechanism of HCQ-induced retinopathy is currently unclear. Accumulation mechanisms may play a role in the development of drug-induced retinopathy.^[20]^ After 4.5 years of intramuscular injection of CQ in rhesus monkeys, the retina and choroid of these experimental monkeys still underwent extensive pathological changes although their appearances and functions were normal in terms of ophthalmology. An analysis of CQ or its byproducts in eye tissues showed that the pigment tissue of the eye had a strong ability to bind to it and accumulation was finally observed in the retina. Similar to CQ, HCQ initially affects ganglion and photoreceptor cells, causing the choroid and pigment epithelium to deteriorate eventually.^[21]^ Recently, bilateral retinal vein occlusion was noted in an HCQ-treated patient, suggesting the possibility of HCQ accumulation.^[22]^

In summary, it was previously believed that more stringent monitoring should be considered in cases of HCQ dose > 5.0 mg/kg, cumulative dose > 1000 g, or renal insufficiency.^[23]^ Typical cases of visual loss in children treated with HCQ have not been reported. This case suggests that in a child with kidney disease using low-dose HCQ, it is necessary to pay close attention to the occurrence of ophthalmic adverse reactions, especially the characteristics of early vision loss. Even though children are treated with low-dose HCQ, the monitoring of HCQ blood concentration should not be ignored. Monitoring of HCQ blood concentration after long-term application should be considered, especially after a sudden decrease in renal function or after dialysis. Increased awareness and early recognition of these symptoms may minimize damage.

## Author contributions

**Conceptualization:** Jinmiao Lu, Feineng Shang, Hong Xu, Zhiping Li.

**Data curation:** Jinmiao Lu, Yidie Huang, Qiaofeng Ye.

**Formal analysis:** Qiaofeng Ye, Mei Ming.

**Funding acquisition:** Zhiping Li.

**Investigation:** Jinmiao Lu, Feineng Shang, Mei Ming, Qiaofeng Ye.

**Methodology:** Jinmiao Lu, Feineng Shang, Qiaofeng Ye.

**Project administration:** Yidie Huang, Hong Xu, Zhiping Li.

**Resources:** Yidie Huang, Hong Xu.

**Software:** Jinmiao Lu, Feineng Shang.

**Supervision:** Hong Xu, Zhiping Li.

**Validation:** Jinmiao Lu, Meiming, Qiaofeng Ye.

**Writing – original draft:** Jinmiao Lu.

**Writing – review & editing:** Yidie Huang, Qiaofeng Ye, Feineng Shang, Mei Ming, Hong Xu, Zhiping Li, Jinmiao Lu.
